# Editorial: “Role of Ribonucleoprotein Complexes in Neurodevelopment and in the Physiopathology of Neurological Diseases”

**DOI:** 10.3389/fmolb.2020.630498

**Published:** 2021-01-29

**Authors:** Carole Gwizdek, Barbara Bardoni, Florence Rage

**Affiliations:** ^1^ Université Côte d’Azur, CNRS, UMR7275, Institut de Pharmacologie Moléculaire et Cellulaire, Valbonne, France; ^2^ Université Côte d’Azur, Inserm, CNRS, UMR7275, Institut de Pharmacologie Moléculaire et Cellulaire, Valbonne, France; ^3^ Institut de Génétique Moléculaire de Montpellier, CNRS-UMR5535, Université de Montpellier, Montpellier, France.

**Keywords:** neurodegenerative disorder, neurodevelopmental disorder, translational regulation, RNA transport, ribonucleoproteic complexes, G-quadruplex, m6A methylation

The topic “Role of Ribonucleoprotein Complexes in Neurodevelopment and in the Physiopathology of Neurological Diseases” is organized in six reviews and two original researches and overall highlights the links existing among the protein components of ribonucleoprotein complexes (RNPs), RNA metabolisms and neuronal disorders.

Neurons are highly polarised cells where mRNA localisation and local protein synthesis underlie the spatio-temporal regulation of protein expression within the axons and dendrites. Many of the RNA-binding proteins (RBPs) controlling mRNA trafficking and local translation are multifunctional factors involved in different steps of mRNA biogenesis (Figures 1A–D). Besides, a single RBP controls a wide variety of mRNAs encoding proteins essential for neuronal development and function. Thus, altered function of those proteins may affect target mRNAs in many different ways. This often results into complex diseases arising from numerous physiopathological mechanisms. In their review, Thelen and Kye recapitulate the role of RBPs in local translation by focusing on four RBPs—Survival Motor Neuron (SMN), Tar DNA binding Protein 43 (TDP-43), Fusion in malignant liposarcoma (FUS) and Fragile X Mental Retardation Protein (FMRP)—known to be involved in severe neurological diseases, such as spinal muscular atrophy (SMA), amyotrophic lateral sclerosis (ALS) and Fragile X syndrome (FXS). They clearly outlined that mutations or deletions of these RBPs prevent the local translation of transcripts within axons or dendrites (Figures 1C,D), leading notably to neuromuscular junction or synaptic connexion impairments (Thelen and Kye). In their article, Sapaly et al. shed light on a common pathway shared by some of these neurodegenerative diseases. Indeed, it has been shown that both TDP-43 and FUS bind SMN protein. Moreover, despite SMN not playing a direct role in ALS, the nuclear Cajal Bodies where SMN in concentrated are significantly reduced in ALS cells. Here, the authors show that the small molecule Flunarizine favours the recruitment of both SMN and TDP-43 in nuclear bodies of SMA cells, suggesting a common action in nucleus of those factors (Figure 1A) (Sapaly et al.). These results are of great interest as they propose that certain drugs could be useful to modulate RNP assemblies in the context of neurodegenerative diseases. As an example, they provide new perspectives in finding efficient treatments stabilizing cytoplasmic SMN-containing neuronal granules in motor neuron pathogenesis, which is an unmet and urgent medical need.

**FIGURE 1:**
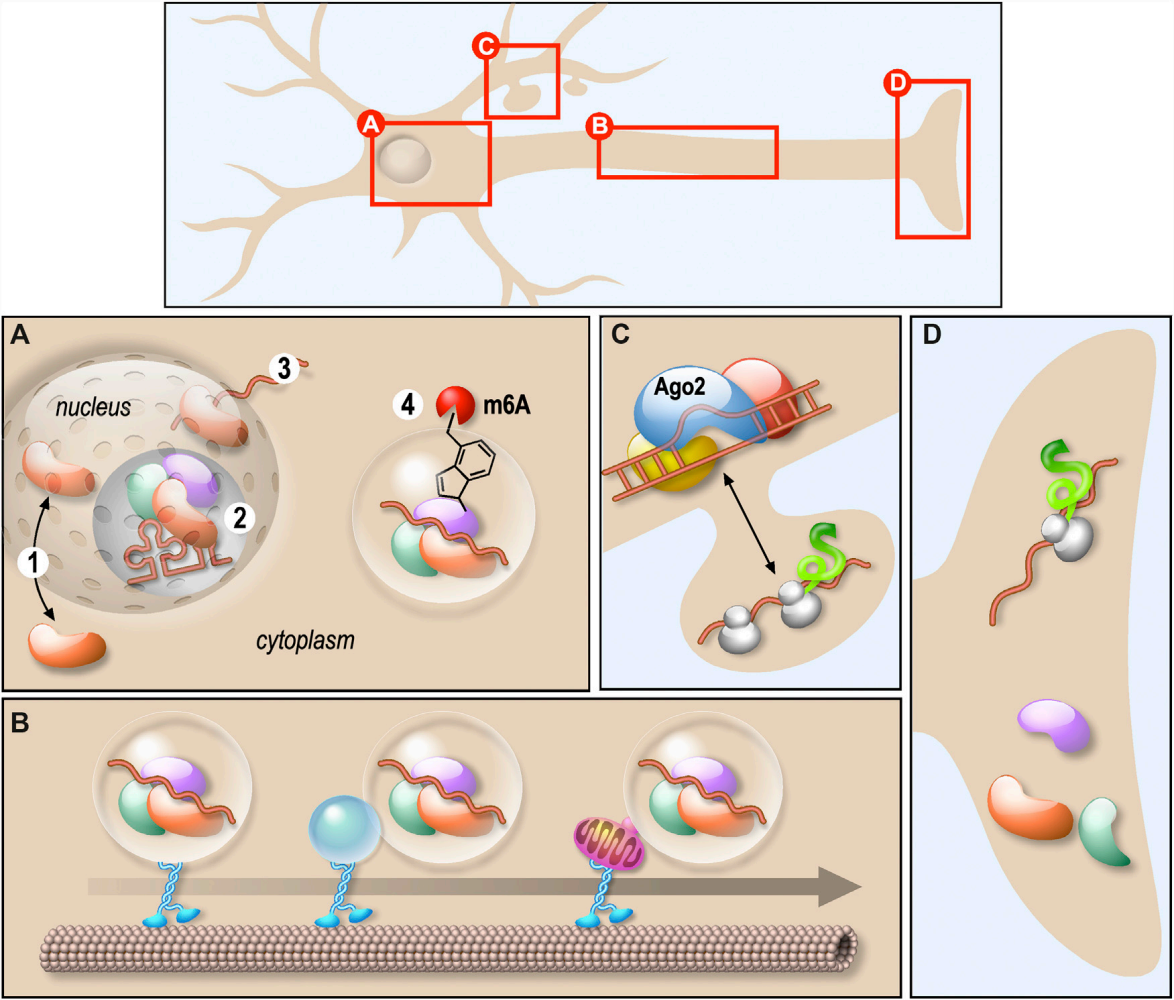
Schematic representation of the role of various RNPs in neurons. **(A)** RNPs biogenesis and shuttling between nucleus and cytoplasm. RNPs shuttle between nucleus and cytoplasm (1), they are involved in several RNA biogenesis steps processing including splicing (2) and nuclear export (3). They also undergo liquid-liquid phase transition regulated by m6A-mediated mRNA (4). **(B)** RNPs transport along dendrites or axons. Membraneless RNPs are composed of RBPs and RNAs associated with motor proteins to travel along microtubules. This association occurs either directly or through membrane-bound organelles. **(C)** RNA degradation. The process of mRNA degradation via miRISC is illustrated. **(D)** Local RNA translation. mRNA transported along axons is locally translated. The translation after transport along dendrites is illustrated in **(C)**.

Interplay between RBPs was also raised in the original research study provided by Imperatore et al. This work is focused on the specific interaction of FUS and FMRP with the G-quadruplex (G4) structures formed in the 3′-UTR of PSD-95 and Shank1a mRNAs, coding for synaptic proteins. The authors suggest that FUS and FMRP could contribute to the regulation of the translation of their common neuronal mRNA targets by competing for the binding to those that harbour G4-forming structures (Figure 1D) (Imperatore et al.).

As described in the review from Dermentzaki and Lotti, RNPs composition may also be regulated by RNA posttranscriptional modifications such as m6A methylation (Figure 1A). Indeed, this modification was recently shown to affect the recruitment of several RBPs on granules thus regulating the stability, the localisation and the translation of the associated mRNAs. In the central nervous system, synapses are preferentially enriched in both m6A-modified transcripts and m6A regulatory proteins. This modification is required for proper neuronal development and function and m6A dysfunctions leads to neurological diseases (Dermentzaki and Lotti).

The fine tuning of protein composition in neurons also relies on the inhibition of translation, either transiently or prior to the degradation of targeted mRNAs. In their review, Nawalpuri et al. underline the diversity of miRNA-induced silencing complex miRISC complexes and how this flexible composition may participate in the control of protein translation (Figure 1C) and, consequently, proper neurogenesis, neuronal migration and differentiation and neural circuits. In particular, they highlight the possible role of miRISC and AGO2 in synaptogenesis and pruning (Nawalpuri et al.)

In the classical view, RNPs behave as membranless granules transported directly along the microtubules via RBPs connecting them to molecular motors. In their review, Pushpalatha and Besse bring some evidence that RNP cargoes may also be anchored to moving organelles that could act as a platform for local translation regulation within the axons (Pushpalatha and Besse). This gives rise to a new level of RNP transport and local translation regulation (Figure 1B).

Last, RBPs can also be connected to the pathophysiology of neurodegenerative diseases as members of pathological aggregates. Loganathan et al. reviewed the recent studies leading to the re-evaluation of the precise mechanisms by which insoluble TDP-43 complexes contribute to many neurodegenerative diseases such as ASL, Frontotemporal dementia or Alzheimer’s (Loganathan et al.). In the case of Fragile X-associated tremor and ataxia syndrome (FXTAS), another neurodegenerative disorder, a major hallmark of the disease is the presence of intranuclear aggregates throughout the brains of FXTAS patients. The pathogenicity derives from the presence of the premutation consisting in the expanded–from 55 to 200–CGG repeats in the 5′ UTR of the gene. In this context, Haify et al. review the *in silico*, *in vitro* and *in vivo* approaches used to attempt to delineate the molecular triggers leading to the neurological phenotypes of the FXTAS pathology (Haify et al.).

This topic gives a rich overview of a research field in fast evolution. The complexity of this field arises from the fact that each RBP targets numerous different mRNAs and is often involved in various steps of their metabolism. In addition, by cross-talking, RBPs participate in different pathogenic mechanisms of neurological disorders which result into common collective phenotypic features. Precisely defining the cellular processes commonly affected in these diseases represents challenging topics for future research. In particular, the identification of common targets of RBPs and their implication in common pathways could result into common therapeutic approaches for neurological disorders due to RNA metabolism deregulation.

## Author Contributions

All authors listed have made a substantial, direct, and intellectual contribution to the work and approved it for publication.

## Conflict of Interest

The authors declare that the research was conducted in the absence of any commercial or financial relationships that could be construed as a potential conflict of interest.

